# Designation of a neotype and redescription of *Hesione reticulata* von Marenzeller, 1879 from Japan (Annelida, Hesionidae)

**DOI:** 10.3897/zookeys.657.11064

**Published:** 2017-02-17

**Authors:** Naoto Jimi, Sergio I. Salazar-Vallejo, Hiroshi Kajihara

**Affiliations:** 1 Department of Natural History Sciences, Graduate School of Science, Hokkaido University, N10 W8, Sapporo 0060-0810, Japan; 2 El Colegio de la Frontera Sur, Depto. Sistemática y Ecología Acuática, Chetumal, México

**Keywords:** Neurochaetal blades, parapodial features, pigmentation pattern, polychaetes, taxonomy

## Abstract

The hesionid polychaete *Hesione reticulata* von Marenzeller, 1879 was described from Enoshima Island, Japan and has been recorded also from the Red Sea. Depending on researchers, it has been regarded as either a distinct species or synonymous with older established ones. The type specimen has been lost. In order to clarify its taxonomic status, *Hesione reticulata* is herein redescribed, illustrated, and a neotype is proposed based on recent material collected near the type locality. The diagnostic features include the presence of several dorsal, discontinuous longitudinal bands, interrupted by pale segmental spots; prostomium with tiny antennae; a tuberculated dorsal integument; acicular lobes double; and neurochaetal blades with guards approaching the distal tooth. The dorsal color pattern in life enables a clear distinction from similar species such as *Hesione intertexta* Grube, 1878 amongst others. Mitochondrial COI barcoding sequences are deposited in the DNA Data Bank of Japan. A key to *Hesione* species from Japan is also included.

## Introduction

The hesionid polychaete *Hesione reticulata* von Marenzeller, 1879 is in need of redescription, in particular incorporating observations of the living pigmentation. This is because delimitation of the species from similar forms, such as *Hesione splendida* Savigny *in* Lamarck, 1818, *Hesione pantherina* Risso, 1826, and *Hesione intertexta* Grube, 1878, involves the color pattern on the dorsal surface, which unfortunately disappears immediately after fixation. Neither the original description (von Marenzeller 1879) nor subsequent redescriptions (Izuka 1912; Imajima and Hartman 1964; Imajima 1997) furnished any illustration that depicts the dorsal color pattern with sufficient quality. Text descriptions on the color pattern, such as “white transverse spots merging to reticulations” (Imajima and Hartman 1964), can refer to a variety of different states, and thus were insufficient to delineate the species and to separate similar forms. Although some color images have been published (Uchida 1992, 2000), their resolution is insufficient for reliable identification.

Further, the morphological features of the currently valid *Hesione* species (after Read and Bellan 2016) have not been standardized nor revised. Dorsal pigmentation can be roughly separated into three patterns: 1) bright grayish in *Hesione splendida* Savigny *in* Lamarck, 1818, 2) transverse bands as in *Hesione genetta* Grube, 1867 and *Hesione picta* Müller, 1858, but in the former there are also round spots along body, and 3) longitudinal lines such as in *Hesione intertexta* Grube, 1878, *Hesione pantherina* Risso, 1826, *Hesione reticulata* von Marenzeller, 1879, and *Hesione steenstrupi* de Quatrefages, 1866. For *Hesione eugeniae* Kinberg, 1866, no pigmentation was given in the description. On the other hand, there are three conditions for the size of neurochaetal guards in comparison with blade’s teeth (Chamberlin 1919:190, Monro 1926:312, 1931:10): approaching the subapical tooth (*Hesione pantherina, Hesione genetta*), approaching the apical tooth (*Hesione intertexta, Hesione reticulata*), and surpassing the apical tooth (*Hesione eugeniae*). More information is needed in order to prepare a key to all species, and this is an expected result of an ongoing revision by one of us (SISV).

The holotype specimen of *Hesione reticulata* was collected by the German naturalist Carl Koerbl on the east coast of Enoshima Island, Kanagawa, during his visit to Japan during 1875–1876. The type material was supposedly deposited in the Natural History Museum in Vienna by Richard von Drasche-Wartinberg (Sato and Sattmann 2009), but is not likely to be extant (Sattmann pers. comm. 2016 email to SISV).

Due to the uncertainties pertaining to some characters in *Hesione reticulata*, the taxonomic status of the species has been doubted by some researchers. Augener (1913) and Hessle (1925) regarded it as synonymous with *Hesione splendida* (type locality: Red Sea); Fauvel (1937: 59) synonymized it with *Hesione pantherina* (type locality: Mediterranean Sea); Wu et al. (1975: 75) viewed it as conspecific with *Hesione intertexta* (type locality: Philippines). Grube (1880: 227) and Hartman (1959:185) regarded *Hesione reticulata* as a distinct species, and von Marenzeller even identified his own species from the Red Sea (Stagl et al. 1996:34). There has been no clarification about the morphological features of *Hesione reticulata* and, by extension, its taxonomic status, and so species delineations are ill-defined.

In this paper, *Hesione reticulata* is redescribed as a distinct species. We designate a neotype as there is no existing type material, and, moreover, there are apparently two different species occurring in Kanagawa Province, which closely resemble each other. Uchida (2009) reported “Hesione cf. ehlersi” and *Hesione reticulata* from Kanagawa, but von Marenzeller’s (1879) original description applies equally well to both of these forms. Uchida (2009: 36) separated these two species in his keys because of differences in pigmentation and chaetotaxy. For *Hesione reticulata*, he indicated yellow dorsal cirrophores, no middorsal reddish brown line, and long blade neurochaetae in chaetigers 1–3, and for Hesione cf. ehlersi pale cirrophores, a middorsal reddish brown line present, and long blade neurochaetae present in chaetigers 1–7. These differences deserve further evaluation because we have noted that, for example, the pigmentation of dorsal cirrophores fades even after being anesthetized, and the presence of long bladed neurochaetae might be size-dependent.

Photographs of the dorsal color pattern in the living state are also provided, as well as of other morphological characters, and the COI barcoding sequence on the basis of freshly-collected material from a place near to the type locality. The key to species of *Hesione* from Japan by Uchida (2009) has been modified and is included below.

## Material and methods

Four specimens were collected at a depth of 1 m by hand in Zaimokuza (35°18'02.9"N, 139°33'02.9"E), Kanagawa Prefecture, Japan. Two specimens were fixed in a 10% formalin sea water solution, later washed and preserved in 70% ethanol (NSMT Pol N-620, NSMT Pol 113205), the other two specimens were fixed and preserved in 70% ethanol (NSMT Pol 113206, NSMT Pol 113207). All specimens were anesthetized with menthol before fixation.

Live and preserved specimens were examined under stereoscopic microscopes (Leica MZ 16F and OLYMPUS BX51); photographs were taken with a digital camera (Nikon D5200). Morphology of chaetae and parapodial features were described from chaetigers 7–9. Neurochaetal blade length was measured from the level of the articulation membrane attachment to chaetal tip; the width was measured at the widest part and expressed as a length:width ratio or by indicating how many times the length corresponds to the width.

Tissue from the dorsal cirri was used for DNA extraction from the two specimens, NSMT Pol N-620 and NSMT Pol 113205. Methods for DNA extraction, PCR amplification, and sequencing followed athose of Jimi et al. (2016). Newly obtained sequences were deposited in DNA Data Bank of Japan (DDBJ) (accession nos. LC169753, LC169754). The neotype and other specimens from the neotype locality, referred to here as paraneotypes (term not regulated by the ICZN 1999) were deposited in the National Museum of Nature and Science, Tsukuba (NSMT), Japan.

## Systematics

### Hesione reticulata

Taxon classificationAnimaliaAciculataHesionidae

von Marenzeller, 1879

Japanese name: otohime-gokai

Figs 1 , 2 , 3

Hesione reticulata von Marenzeller, 1879: 129–131, pl. 3, fig. 4; Izuka 1912: 192–194, pl. 2, fig. 7; Imajima and Hartman 1964: 80; Uchida 2009: 36–37, fig. 1.
Hesione splendida Hessle 1925: 13–15 (*non* Savigny *in* Lamarck, 1818; *partim*, smallest specimen with transverse white bands belongs elsewhere).
Hesione pantherina Fauvel 1937: 59–60 (*non* Risso, 1826).
Hesione intertexta Wu et al. 1975: 75, pl. 2, figs 7–8 (*non* Grube, 1878).

#### Type material.

**Northwestern Pacific, Japan**. Neotype NSMT Pol N-620, and three paraneotypes NSMT Pol 113205, NSMT Pol 113206, NSMT Pol 113207, Zaimokuza (35°18'02.9"N, 139°33'02.9"E), rocky bottom, 1 m depth, 19 Mar. 2016, N. Jimi & Hesione Tanaka, coll. Paraneotypes (NSMT Pol 113205 – NSMT Pol 113207) 40–47 mm long, 4 mm wide).

#### Neotype locality.

Zaimokuza (35°18'02.9"N, 139°33'02.9"E), rocky bottom, 1 m depth.

#### Description.

**Figure 1.**
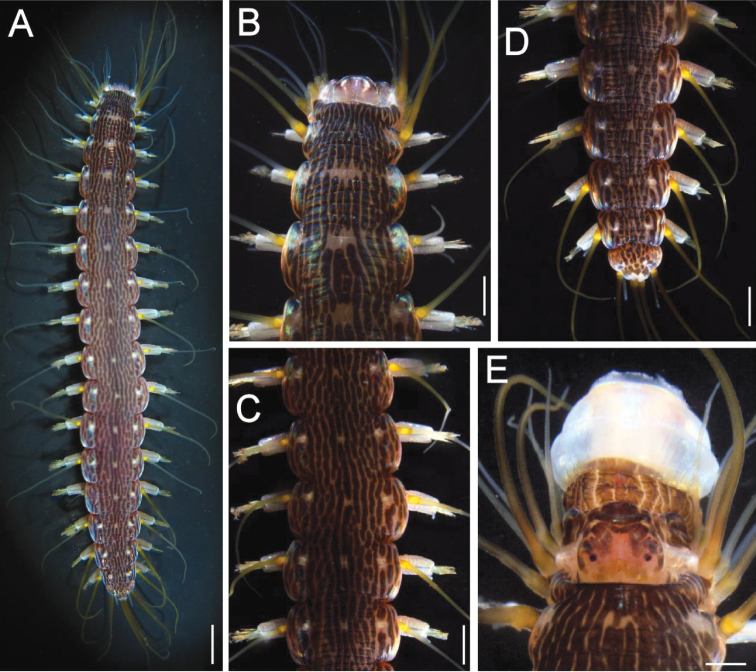
*Hesione reticulata* von Marenzeller, 1879, anesthetized living specimens. **A–D** (NMST Pol N-620) **E** (NSMT Pol-113206). **A** Dorsal view **B** Anterior end, dorsal view **C** Medial part **D** Posterior end, dorsal view **E** anterior end, dorsal view. Scale bars: 3.2 mm (**A**); 1.1 mm (**B–D)**; 0.7 mm (**E**).

Neotype (NSMT Pol N-620) complete. Body cylindrical, medially swollen (Fig. 1A), damaged, 43 mm long, 4 mm wide in chaetigers 8–9 (not including parapodia), 16 chaetigers (chaetae and parapodia of 2^nd^ left, 8^th^ right, and 9^th^ right chaetigers removed for observation; dorsal cirri of 3^rd^ and 7^th^ chaetigers removed for DNA extraction).

**Figure 2.**
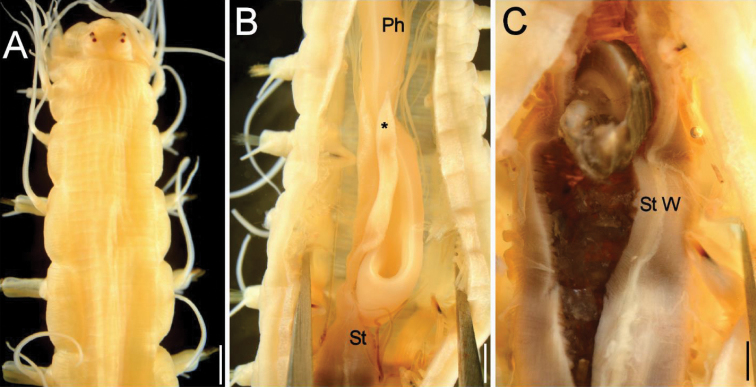
*Hesione reticulata* Marenzeller, 1879, **A** (NSMT Pol N-620) **B–C** (NSMT Pol-113205) **A** Anterior end, dorsal view, six months after fixation **B** Medial part, ventral view after longitudinal dissection (*: enteric caecum, Ph: pharynx, St: stomach) **C** Same, close up after dissecting stomach, with a gastropod prey (St W: stomach wall). Scale bars: 1.5 mm (**A**); 1.2 mm (**B**); 0.6 mm (**C**).

*Dorsal pigmentation pattern* consisting of longitudinal, brownish, subcontinuous, irregular lines; no reddish brown longitudinal broken line on median line; single, irregularly-shaped spot (formed by absence of brown pigment, through which basement pale tan to wheat body color seen) on each chaetiger except 2nd, arranged mid-dorsally (larger anteriorly; reduced medially and posteriorly); and additional row of similar but smaller spots on lateral cushion on each side; silvery white spots absent. Cirrophores yellow; cirrostyles yellow to whitish; parapodial lobes whitish (Fig. 1B–E). After six months in ethanol, pigmentation limited to dorsal, pale brown, discontinuous longitudinal bands (Fig. 2A).

*Integument* smooth, annulated, giving impression of being tuberculated, especially along posterior region; longitudinal ridges absent in lateral cushions.

*Prostomium* heart-shaped, wider than long (Fig. 1B, E); anterior margin truncated; lateral margins rounded in anterior body, but expanded posteriorly; posterior margin cleft, as long as 1/6 prostomial length; longitudinal furrow shallow; dark transverse line present on prostomial anterior margin. Antennae digitate, twice longer than wide. Eyes blackish, on center of prostomium; anterior and posterior eyes in trapezoidal arrangement; anterior eyes slightly more separated than posterior eyes; anterior eyes ovoid (appearing longer than wide), posterior eyes rounded.

*Tentacular cirri* tapered, longest complete anterior cirri reaching chaetiger 5. Lateral cushions slightly projected, entire, with smooth surface.

**Figure 3.**
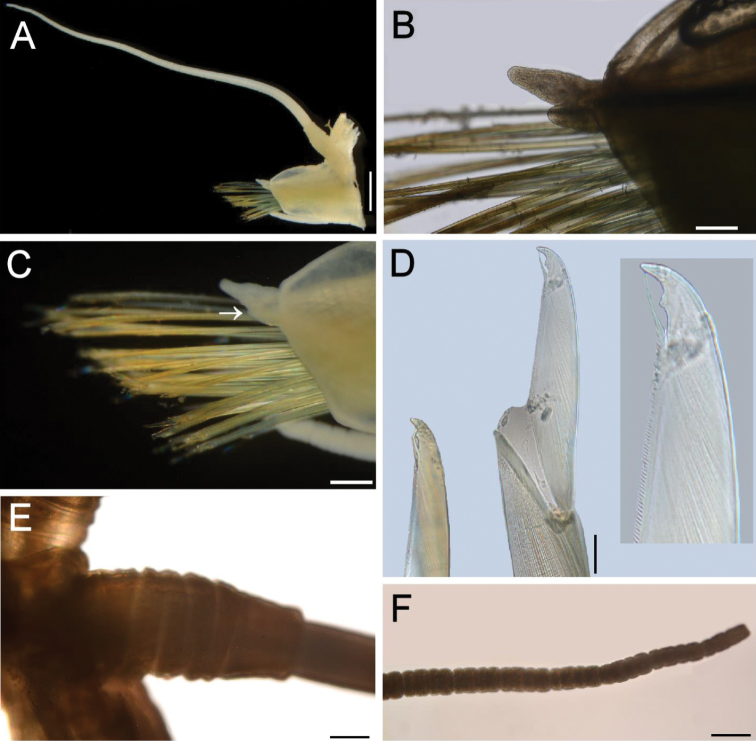
*Hesione reticulata* Marenzeller, 1879, **A–F** (NSMT Pol N-620). **A** Chaetiger 8, right parapodium, anterior view **B** Chaetiger 9, right parapodium, anterior view, close-up of acicular lobe **C** Chaetiger 8, right parapodium, anterior view, close-up of acicular lobe (arrow points to lower tine) and neurochaetal bundle **D** Same, tip of neurochaetae (inset: blade tip) **E** Same, close-up of dorsal cirrophore **F** Same, close-up of cirrostyle distal region. Scale bars: 0.78 mm (**A)**; 0.15 mm (**B**); 0.17 mm (**C**), 25 μm (**D**); 0.13 mm (**E, F**).

*Parapodia* with dorsal cirrophore twice longer than wide, articulated (Fig. 3A, E). Cirrostyle basally cylindrical, medially and distally articulated, as long as body width, including parapodia (Fig. 3A, F). Neuropodia with parallel sides, cylindrical (Fig. 3A). Acicular lobe double; upper tine twice larger than lower one, digitate (Fig. 3B); lower tine of 8^th^ chaetiger of NSMT Pol N-620 and 9^th^ chaetiger of NSMT Pol 113205 adhered or fused to upper tine and difficult to observe (Fig. 3C), it can be clearly confirmed on other four parapodia examined. One acicula present, blackish. Neurochaetae 19–28 per bundle, blade size decreasing ventrally (Fig. 3C); neurochaetal blades bidentate, 3–4 times longer than wide, subdistal tooth shorter and wider than distal one; guard reaching apical tooth (Fig. 3D). Ventral cirrophore three times wider than long; cirrostyle articulated, surpassing chaetal lobe tip.

*Prepygidial segment* with two cirri, three times as long as body width of previous chaetiger (chaetiger 16). Pygidium smooth, trapezoidal, as long as wide, cylindrical (Fig. 1D); anus with two anal cirri; anal cirri tapered.

*Venter* without pigmentation, with longitudinal midventral depression.

*Pharynx* divided into three rings, with relative lengths 1.5:1.5:1; basal ring with similar pigmentation as anterior end (Fig. 1E); dorsal papilla pale, longer than wide (length: width 1.5:1).

*Oocytes* not visible.

#### Inner anatomy

**(observed in paraneotypes NSMT Pol 113205–113207).** Pharynx and esophagus thick, muscular, yellowish, continuing into darker, shorter stomach; enteric caeca extending anteriorly along two or three chaetigers (Fig. 2B). Stomach contents included gastropod (Fig. 2C) and amphipod remains as prey items.

#### Remarks.

The nomenclatural status of *Hesione reticulata* von Marenzeller, 1879 has been unclear due to several reasons: there is no type material, some diagnostic features were not clarified in the original description, the species has been recorded from the Red Sea, and some authors have regarded it as a junior synonym of other species within the genus. Consequently, in order to comply with the International Code of Zoological Nomenclature (ICZN 1999, Art. 75.3) we are herein proposing a neotype.

The above description and illustrations will clarify the taxonomic status of *Hesione reticulata* (Art.75.3.1), and its diagnostic and differential features have been included in the description and illustrations (Arts 75.3.2, 75.3.2), and will be contrasted below. Our enquiries on the existence of type material of *Hesione reticulata* to Dr. Helmut Sattmann, Curator of Marine Invertebrates, in the Naturhistorisches Museum, Vienna, where Emil von Marenzeller used to work and produced all of his publications, indicate that type material is absent (Art. 75.3.4), probably destroyed or never deposited. The original collector was Carl Koerbl (von Marenzeller 1879: 131) and some of his specimens were donated to the Vienna Museum by Richard von Drasche-Wartinberg (Sato and Sattmann 2009), but there is no type material available there.

The neotype fits the original description and because it was recently collected, it even matches the general pigmentation pattern which is not long-lasting in ethanol. Further, as happens in some other species of *Hesione*, they are simultaneous hermaphrodites (Bergmann 1902, 1903), so that differences in pigmentation pattern among different specimens cannot be attributed to sex (Art. 75.3.5). Thus, the morphology of our material does not contradict von Marenzeller’s (1879) original description of *Hesione reticulata*, nor the general features subsequently described by Izuka (1912: 192), Imajima and Hartman (1964: 80), and Uchida (2009: 36–37).

The original specimen was collected in the east coast of Enoshima (35°18'07"N, 139°29'00"E), and the neotype was found in Zaimokuza (35°18'02.9"N, 139°33'02.9"E), nearly four kilometers away, such that we are confident these two localities belong to the same ecological unit (Art. 75.3.6). The neotype of *Hesione reticulata* has been deposited in the National Science Museum, Tokyo, which holds the most important polychaete collection in Japan, and has a very important tradition in the scientific study of polychaetes from Japan and elsewhere (Art. 75.3.7).

Another taxonomic relevance of our study lies in the identification of the dorsal color pattern in the living state as a clear distinguishing feature between *Hesione reticulata*, *Hesione intertexta*, and Hesione cf. ehlersi sensu Uchida (2009). The color pattern agrees with von Marenzeller’s (1879) description of the holotype which had, over a reddish-brown background, irregular spots fused into wide bands along some anterior segments continuing to the end of the body. von Marenzeller (1879) mostly relied on this complex reticulated pigmentation pattern for justifying the establishment of *Hesione reticulata*.

Ngamniyom et al. (2014) and Lee and Ong (2015) characterized the two western Pacific species, Hesione cf. picta and *Hesione intertexta*. The former has wide dorsal transverse bands, by which Hesione cf. picta can be separated from *Hesione intertexta* and *Hesione reticulata*, because the latter two have dorsal, longitudinal, discontinuous dark bands with paler spots mid-dorsally and along dorsal surface of lateral cushions. Furthermore, *Hesione intertexta* and *Hesione reticulata* also have tiny antennae and neurochaetal blades with guards approaching distal tooth. Based on these shared characteristics, Wu et al. (1975) viewed *Hesione reticulata* as a junior synonym of *Hesione intertexta*. Our observation, however, clearly shows that they differ in pigmentation pattern: in *Hesione reticulata* the paler spots are smaller, and the mid-dorsal ones tend to be round, whereas in *Hesione intertexta* they are longer than wide and markedly larger.

Uchida (2009) described Hesione cf. ehlersi, a species with similar morphological features to *Hesione reticulata*. Indeed, von Marenzeller’s original description of *Hesione reticulata* could apply to both species. As Uchida (2009) stated, dorsal pigmentation in life is useful for discrimination of the two species; Hesione cf. ehlersi has a reddish brown longitudinal broken line on the median line, whereas *Hesione reticulata* lacks this line. Further study is needed to resolve the taxonomic position of Hesione cf. ehlersi.

The vivid images of the dorsal color pattern in *Hesione reticulata*, along with the COI barcoding sequence provided in this paper, will contribute to future taxonomic revision of the genus *Hesione*.

*Hesione reticulata* was regarded as a distinct species by Hartman (1959: 185) and it can be distinguished from its former synonyms *Hesione intertexta*, *Hesione splendida* as indicated by Augener (1913) and Hessle (1925), or from *Hesione pantherina* as suggested by Fauvel (1937) as follows: from *Hesione splendida*, *Hesione reticulata* can be separated by the dorsal pigmentation; it is brownish in *Hesione reticulata*, but pearly gray in *Hesione splendida* (Savigny, 1822), whereas from *Hesione pantherina*, *Hesione reticulata* can be distinguished because the guard tooth in *Hesione reticulata* reaches the apical tooth, whereas those in *Hesione pantherina* do not (Monro 1926).

One of the important discoveries in our observation of the specimens of *Hesione reticulata* is that the acicular lobe in this species is doubled, comprised of the upper and lower tines, a character state that separates *Hesione* species in two groups, each with approximately the same number of species (SISV pers. obs.). von Marenzeller (1879, fig. 4) illustrated a parapodium excised from the middle part of the body in the holotype specimen, indicating that there was a single, thick, finger-shaped acicular lobe, unlike the doubled lobe that we observed in this study. Izuka (1912) and Imajima and Hartman (1964) also described the acicular lobe as a single lobe. In two of the six parapodia examined (left one on the 2^nd^, right one on the 8^th^, and right one on the 9^th^ chaetigers from NSMT Pol N-620; right one on the 9^th^ chaetiger from NSMT Pol 113205; and left ones on the 3^rd^ and 9^th^ chaetigers from NSMT Pol 113207), the lower tine adhered to the upper tine. It appeared as if it were a single parapodial lobe, but a careful observation showed that it actually represents a doubled lobe. The reason the acicular lobe was described as ‘single’ in the previous studies may be that the lower tine in their material was deformed in preservation to lie below the upper tine, or to contact closely to the upper tine. The original illustration (von Marenzeller 1879, fig. 4) clearly indicates that the acicular lobe was placed under the chaetal bundles on the glass slide. This must have made the acicular lobe difficult to be observed, which would also explain why the adjacent upper and lower tines were hardly detected. This feature further adds to the distinction between *Hesione reticulata* and *Hesione intertexta*: the acicular lobe in *Hesione reticulata* is double whereas it is single in *Hesione intertexta*.

The record of *Hesione reticulata* by Imajima (1997: 171) might not belong to the same species because he indicated that the acicular lobe was single (“a superior conical papilla”), and because unlike our specimens, his material was collected from 230–250 m depth in Suruga Bay. Other specimens recorded as *Hesione reticulata* by Imajima (2003: 132–134), collected in shallow water, were characterized as having acicular lobe single (“a superior conical papilla”), and are regarded as belonging to another species.

**Distribution.** *Hesione reticulata* has so far been recorded only from Japan: Kanagawa (von Marenzeller 1879; Izuka 1912; this study), Shizuoka and Wakayama (Izuka 1912), and the middle of Honshu to Kyushu (Uchida 2009).

##### Key to species of *Hesione* from Japan

(modified from Uchida 2009)

**Table:** 

1	Antennae present; eyes positioned centrally on prostomium	**2**
–	Antennae absent; eyes displaced anteriorly	***Hesione* ? sp.**
2	Neurochaetal blades with guard	**3**
–	Neurochaetal blades without guard	**Hesione splendida ? sensu Monro, 1931**
3	Dorsum with transverse bands; neurochaetal guards approaching subdistal tooth	**4**
–	Dorsum with longitudinal bands; neurochaetal guards approaching distal tooth	**5**
4	Chaetiger 2 pale; dorsal pigmentation without spots; second tentacular cirri markedly longer than fourth	***Hesione genetta* Grube, 1867**
–	Chaetiger 2 with a black band; dorsal pigmentation includes spots; second tentacular cirri as long as fourth	***Hesione* sp.**
5	Longitudinal bands short; silvery white spot present mid-dorsally	***Hesione intertexta* Grube, 1878**
–	Longitudinal bands long; silvery white spot absent	**6**
6	Dorsal cirrophores pale; neurochaetae with long blades in chaetigers 1–7; acicular lobe as long as chaetal lobe width; mid-dorsal reddish brown, subcontinuous line present	***Hesione cf. ehlersi***
–	Dorsal cirrophores yellow; neurochaetae with long blades in chaetigers 1–3; acicular lobe shorter than chaetal lobe width; mid-dorsal reddish brown line absent	***Hesione reticulata* von Marenzeller, 1879**

## Supplementary Material

XML Treatment for Hesione reticulata

## References

[B1] Augener H (1913) Polychaeta I, Errantia. Die Fauna Südwest – Australiens 4(5): 63–304. [pls 2–3] http://biodiversitylibrary.org/page/7160888

[B2] Bergmann W (1902) Untersuchungen über die Eibildung bei Anneliden und Cephalopoden. Zeitschrift für wissenschaftliche Zoologie 73: 278–301. [pls 17–19] http://biodiversitylibrary.org/page/43239588

[B3] Bergmann W (1903) Über das spätere Schicksal der Zwitterdrüssen von *Hesione sicula*. Zoologischer Anzeiger 26: 415–417. http://biodiversitylibrary.org/page/30125385

[B4] Chamberlin RV (1919) The Annelida Polychaeta of the Albatross Tropical Pacific Expedition, 1891–1905. Memoirs of the Museum of Comparative Zoology of Harvard College 31: 1–514. http://dx.doi.org/10.5962/bhl.title.49195

[B5] de Quatrefages A (1866) Histoire Naturelle des Annéles marins et d’Eau Douce. Annélides et Gephyriens. Librarie Encyclopèdique de Roret, Paris, volume 1, 588 pp.

[B6] Fauvel P (1937) Annélides polychètes du Japon. Memoirs of the College of Science, Kyoto Imperial University, Series B 12 [1936]: 41–92.

[B7] Grube AE (1867) ) Neue Anneliden aus den Gattungen Eunice, Hesione, Lamprophaës und Travisia. Jahres–Bericht der Schlesischen Gesellschaft für vaterländische Cultur 44[1866]: 64–66. http://biodiversitylibrary.org/page/46548206

[B8] Grube AE (1878) Annulata Semperiana. Beiträge zur Kenntniss der Annelidenfauna der Philippinen. Memoires de l’Academie Imperiale des Sciences de St. Petersbourg, Septième Série 25(8): 1–300. http://dx.doi.org/10.5962/bhl.title.85345

[B9] Grube AE (1880) Mittheilungen über die Famile der Phyllodoceen und Hesioneen. Jahresbericht der Schlesischen Gesellschaft für vaterländische Cultur 57: 204–228. http://biodiversitylibrary.org/page/46547075

[B10] Hartman O (1959) Catalogue of the Polychaetous Annelids of the World. Allan Hancock Foundation Publications, Occasional Paper 23: 1–628. http://digitallibrary.usc.edu/cdm/ref/collection/p15799coll82/id/19573

[B11] Hessle C (1925) Einiges über die Hesioniden und die Stellung der Gattung *Ancistrosyllis*. Arkiv för Zoologi 17: 1–36.

[B12] Imajima M (1997) Polychaetous annelids of Suruga Bay, Central Japan. National Science Museum Monographs (Tokyo) 12: 149–228. http://ci.nii.ac.jp/els/110004312462.pdf?id=ART0006480764&type=pdf&lang=en&host=cinii&order_no=&ppv_type=0&lang_sw=&no=1476474983&cp=

[B13] Imajima M (2003) Polychaetous annelids from Sagami Bay and Sagami Sea collected by the Emperor Showa of Japan and deposited at the Showa Memorial Institute, National Science Museum, Tokyo, 2. Orders included within the Phyllodocida, Amphinomida, Spintherida and Eunicida. National Science Museum Monographs 23: 1–221. http://ci.nii.ac.jp/naid/110004708004/en

[B14] Imajima M Hartman O (1964) The polychaetous annelids of Japan, 1. Allan Hancock Foundation Publications, Occasional Paper 26: 1–166. http://cdm15799.contentdm.oclc.org/cdm/ref/collection/p15799coll82/id/18946

[B15] ICZN (1999) International Code of Zoological Nomenclature (4^th^ edn). International Trust for Zoological Nomenclature, London.

[B16] Izuka A (1912) The errantiate Polychaeta of Japan. Journal of the College of Science (Tokyo) 30: 1–262. http://hdl.handle.net/2261/32884

[B17] Jimi N Tanaka M Fujiwara Y (2016) *Diplocirrus nicolaji* (Annelida: Flabelligeridae) from Japan, detailed morphological observation and DNA barcoding. Marine Biodiversity Records 9(1): 1–8. https://doi.org/10.1186/s41200-016-0024-7

[B18] Kinberg JGH (1866) Annulata nova (Nephthydea, Phyllodocea, Alciopea, Hesionida, Gycerea, Goniadea, Syllidea, Ariciea, Spiodea, Aonidea, Cirratulida, Opheliacea). Öfversigt af Kongelige Vetenskaps–Aakademiens Förhandlingar 22[1865]: 239–258. http://biodiversitylibrary.org/page/32339515

[B19] Lamarck JBPA de (1818) Histoire naturelle des animaux sans vertèbres, présentant les caractères généraux et particuliers de ces animaux, leur distribution, leurs classes, leurs familles, leurs genres, et la citation des principales espèces qui s’y rapportent; précédés d’une introduction offrant la détermination des caractères essentiels de l’animal, sa distinction du végétal et des autres corps naturels, en fin, l’exposition des principes fondamentaux de la zoologie, Vol. 5. Deterville & Verdiere, Paris, 612 pp http://dx.doi.org/10.5962/bhl.title.12712

[B20] Lee Y Ong R (2015) New records of two hesionid polychaetes from the Singapore Strait. Singapore Biodiversity Records 2015: 201–204. https://lkcnhm.nus.edu.sg/nus/images/pdfs/sbr/2015/sbr2015-201-204.pdf

[B21] Monro CCA (1926) Polychaeta of the ‘Alert’ Expedition. Families Hesionidae and Nereidae. Journal of the Linnean Society of London, Zoology 36(243): 311–323. https://doi.org/10.1111/j.1096-3642.1926.tb02172.x

[B22] Monro CCA (1931) Polychaeta, Oligochaeta, Echiuroidea, and Sipunculoidea. Great Barrier Reef Expedition 1928–29, Scientific Reports 4: 1–37. http://biodiversitylibrary.org/page/49516539

[B23] Müller F (1858) Einiges über die Annelidenfauna der Insel Santa Catharina an der brasilianischen Küste. Archiv für Naturgeschichte 24: 211–220. [pls 6–7] http://biodiversitylibrary.org/page/7460059

[B24] Ngamniyom A Silprasit K Sriyapai T (2014) Morphological and molecular evidence for a new record of Hesione cf. picta (Polychaeta: Hesionidae) from the Western coast of the Gulf of Thailand. Kasetsart Journal, Natural Sciences 48: 719–728. http://www.thaiscience.info/journals/Article/TKJN/10961404.pdf

[B25] Read G Bellan G (2016) *Hesione* Savigny *in* Lamarck, 1818 In: Read G, Fauchald K (Eds) World Polychaeta Database. http://www.marinespecies.org/aphia.php?p=taxdetails&id=129308 [Jan. 7, 2017]

[B26] Risso A (1826) Histoire naturelle des principales productions de l’Europe méridionale et particulièrement de celles des environs de Nice et des Alpes maritimes, Vol. 4. F.G. Levrault, Strasbourg, 439 pp http://biodiversitylibrary.org/page/39428699

[B27] Sato M Sattmann H (2009) Extirpation of *Hediste japonica* (Izuka, 1908) (Nereididae, Polychaeta) in Central Japan, evidenced by a museum historical collection. Zoological Science 26: 369–372. https://doi.org/10.2108/zsj.26.369 19715508 10.2108/zsj.26.369

[B28] Savigny JC (1822) Système des annélides, principalement de celles des côtes de l’Égypte et de la Syrie, offrant les caractères tant distinctifs que naturels des ordres, familles et genres, avec la description des espèces. Description de l’Egypte. Histoire naturelle, Paris 21: 1–128. http://dx.doi.org/10.5962/bhl.title.66284

[B29] Stagl V Sattmann H Dworschak PC (1996) The material of the *Pola* Red Sea expeditions (1895–1898) in the collections of the Natural History Museum in Vienna. Biosystematics and Ecology Series 11: 29–41. http://www.nhm-wien.ac.at/jart/prj3/nhm/data/uploads/mitarbeiter_dokumente/dworschak/Stagl_et_al1996_Pola.pdf

[B30] Uchida H (1992) [Annelida, Polychaeta In: Nishimura S (Ed.) Guide to Seashore Animals of Japan with Color Pictures and Keys (Vol. 1). 310–373. [pls 63–72, In Japanese]

[B31] Uchida H (2000) [Animals of Chiba Prefecture 2, Marine Animals of Chiba Prefecture: Annelida]. In: The Foundation of Chiba Prefecture for the Study of Historical Materials (Ed.) Natural History of Chiba Prefecture 7. The Foundation of Chiba Prefecture for the Study of Historical Materials, Chiba, 278–291. [In Japanese]

[B32] Uchida H (2009) [Polychaetologica 55: Key to genus and description of species (40), Hesionidae 4]. Marine Pavilion, Kushimoto Marine Park 38: 36–37. [In Japanese]

[B33] Von Marenzeller E (1879) Sudjapanische Anneliden, 1. Amphinomea, Aphroditea, Lycoridea, Phyllodocea, Hesionea, Syllidea, Eunicea, Glycerea, Sternaspidea, Chaetopterea, Cirratulea, Amphictenea. Denskschriften der Mathematisch-Naturwissenschaftlichen classe der Kaiserlichen Akademie der Wissenschaften 41: 109–154.

[B34] Wu B Shen S Chen M (1975) [Preliminary report of polychaetous annelids from Xisha Islands, Guangdong Province, China]. Studia Marina Sinica 10: 65–104.

